# The-state-of-the-art of soft robotics to assist mobility: a review of physiotherapist and patient identified limitations of current lower-limb exoskeletons and the potential soft-robotic solutions

**DOI:** 10.1186/s12984-022-01122-3

**Published:** 2023-01-30

**Authors:** Leah Morris, Richard S. Diteesawat, Nahian Rahman, Ailie Turton, Mary Cramp, Jonathan Rossiter

**Affiliations:** 1 Centre for Health and Clinical Research, University of the West of England, Bristol, UK; 2 Bristol Robotics Laboratory, Bristol, UK; 3 Department of Engineering Mathematics, University of Bristol, Bristol, UK

**Keywords:** Mobility, Rehabilitation, Patient, Therapist, Exoskeleton, Assistive device, Soft robotics

## Abstract

**Background:**

Soft, wearable, powered exoskeletons are novel devices that may assist rehabilitation, allowing users to walk further or carry out activities of daily living. However, soft robotic exoskeletons, and the more commonly used rigid exoskeletons, are not widely adopted clinically. The available evidence highlights a disconnect between the needs of exoskeleton users and the engineers designing devices. This review aimed to explore the literature on physiotherapist and patient perspectives of the longer-standing, and therefore greater evidenced, rigid exoskeleton limitations. It then offered potential solutions to these limitations, including soft robotics, from an engineering standpoint.

**Methods:**

A state-of-the-art review was carried out which included both qualitative and quantitative research papers regarding patient and/or physiotherapist perspectives of rigid exoskeletons. Papers were themed and themes formed the review’s framework.

**Results:**

Six main themes regarding the limitations of soft exoskeletons were important to physiotherapists and patients: safety; a one-size-fits approach; ease of device use; weight and placement of device; cost of device; and, specific to patients only, appearance of the device. Potential soft-robotics solutions to address these limitations were offered, including compliant actuators, sensors, suit attachments fitting to user’s body, and the use of control algorithms.

**Conclusions:**

It is evident that current exoskeletons are not meeting the needs of their users. Solutions to the limitations offered may inform device development. However, the solutions are not infallible and thus further research and development is required.

**Supplementary Information:**

The online version contains supplementary material available at 10.1186/s12984-022-01122-3.

## Introduction

In the UK, 6.8 million people live with mobility-related disabilities; the leading causes of which are musculoskeletal conditions and stroke [1, 2]. Persons with stroke are living longer due to reductions in risk factors and improvements of treatments [2]. The population overall is also aging; the number of people living over 85 is expected to increase from 1.8 million in 2018 to 3 million by 2043 [3]. Musculoskeletal impairments are associated with older age, therefore both those with musculoskeletal impairments and stroke survivors are living longer with disabilities that require assistance [2, 4].

Impaired mobility can have widespread effects on an individual’s quality of life as participation challenges impact their work, social life and activities of daily living (ADLs) [5]. Mobility impairments are also a risk factor for falls which reduce an individual’s confidence and self-belief in their own mobility, and can lead to activity avoidance, social isolation and depression, which in turn increases frailty and the ‘fear of falling’ cycle [6–8]. Thus, the paramount goal for physiotherapy rehabilitation is to ensure the continued mobility of individuals, with evidence demonstrating that, for neurological patients, repetitive movements are crucial to re-learn motor functions [9]. This is not without its challenges; in the UK, persons with stroke typically receive only 35 minutes of inpatient physiotherapy per day, despite the guidance of 45 minutes minimum [10, 11]. Increasing rehabilitation time may not be achievable as traditional rehabilitation frequently requires body weight support of the patient, which can be physically demanding for the physiotherapist who may require assistance from others [12, 13]. Consequently, therapist fatigue and staffing capacity limits what a patient is able to achieve in a session [13]. Assistive devices such as walkers are commonly provided to patients with mobility impairments [14]. These devices fall under the umbrella of ’assistive technology’, which describes products or systems that assist individuals with disabilities, restricted mobility or other impairments to perform functions that might otherwise be impossible or challenging [15]. Although assistive devices can improve rehabilitation of muscle and neural processing, they have limitations that prevent individuals from carrying out their ADLs as normal [14]. Reported challenges include opening of doors or getting on to public transport when using four-wheeled walkers, and issues carrying items, food and drink when using a walking stick [14, 16, 17].

Development in wearable powered exoskeletons offers a potential solution to traditional rehabilitation challenges [18, 19]. An exoskeleton, also known as a wearable robot, is a mechanical system worn by humans to augment, complement or substitute the function of the wearer’s limbs [20]. Early developed exoskeletons were stationary devices used to train patients on a treadmill with body-weight support, reducing loads on lower limbs for rehabilitation, such as DGO [21], LOPES [22] and ALEX [23]. Later, commercially available, portable assistive exoskeletons were developed, including Ekso, Rewalk [24], Indego and Exo H2 with an increasing number in development [12, 25]. Although not identical, their principles and designs are similar, consisting of an external actuator(s) fitted in parallel with weak or paralysed lower limbs to assist with mobilising and activities of daily living [26].

Many of these existing rigid exoskeletons were initially developed to provide maximal assistance to those with complete paralysis resulting from spinal cord injury. Interest has increased in the exoskeletons that can provide sensory-guided motorised lower limb assistance for person’s with stroke [27, 28]. These devices provide partial assistance during mobility tasks, allowing persons with stroke to actively participate through practising postural control and locomotion patterns [12]. A systematic review with a meta-analysis demonstrated that rigid exoskeletons are safe, with no reported adverse events, with falls only reported in a study using an early prototype [26, 29]. Further, rigid exoskeletons have widespread benefits including increased walking time, number of steps and improved strength and postural control in stroke survivors [30]. Studies have only recently explored patients and physiotherapists’ perspectives of the use of exoskeletons [18, 25, 31]. A key advantage of existing rigid exoskeletons was their ability to reduce the physical strain on therapists, therefore fewer members of staff would be needed to assist a patient, increasing the service’s capacity [18, 31]. The ways in which exoskeletons may have psychosocial benefit to individuals was also highlighted, including the potential improvement to a patient’s confidence and feeling of independence [18, 31].

Despite these proposed advantages, rigid exoskeletons have not been widely adopted clinically [32]. Although a systematic literature review on user perspective of rigid exoskeletons has been undertaken previously by Hill et al. [33], the review only included three papers which had limited reporting of qualitative data and their methods were predominantly quantitative components [29, 34, 35]. The review was inconclusive on user perspectives of rigid exoskeletons due to the minimal amount of evidence that has been undertaken; nevertheless, they concluded that users are able to offer their opinions, which may facilitate the design process. Since the publication of the Hill et al. [33] review, there has been further research into patient and physiotherapists’ perspectives; papers highlighted a range of rigid exoskeleton limitations, and they also recognised their novelty and potential [18, 25, 31]. Common perceived limitations or concerns regarding rigid exoskeletons across studies included: safety issues such as joint misalignment; creation of only one device to fit all patients; difficulty of use, including donning and doffing; weight and cost; and device appearance [18, 25, 31, 36]. User perspectives for traditional rigid exoskeletons demonstrated a disconnect between those with clinical knowledge who understand the requirements of assistive devices, and the engineers with the technical knowledge to create such devices [25].

Soft-robotics is an emerging field with capabilities to address the limitations of bulky, rigid and heavy exoskeletons through designing wearable, soft devices that are lightweight, compliant and flexible, resulting in safe human interaction, suitable for body assistance [37, 38]. Soft exoskeletons are different from rigid exoskeletons as they have an interface with the wearer that is a non-rigid structure, for instance, textiles, velcro or straps [39]. A device may also be a hybrid of both, and would therefore not technically be a fully soft exoskeleton (despite sometimes being described as a soft exoskeleton). Examples include an exoskeleton with compliant/soft actuators but a rigid structure-based body attachment, or a system with rigid actuators mounted on the body using a soft structure. A review of 52 lower-limb exoskeletons found that only 11% were fully soft exoskeletons [40]. A recent review on soft wearable robots reported an exponential growth of using pneumatic artificial muscles (PAMs), electrically-driven actuators, and textiles/fabric-based actuators over the past 10 years [41].

If the advantages of soft robotics are to be realised and implemented effectively and transitioned rapidly into clinical and community settings, the engineering and clinical gap must be reduced. Consequently, this state-of-the-art review aims to identify the limitations of existing exoskeletons as perceived by clinicians and patients, and discuss the potential soft-robotic solutions. The review informs FREEHAB, a project which aims to design wearable, assistive soft-robotic devices for people with impaired mobility (project website - The Right Trousers [42]). This collaborative review was undertaken by Freehab researchers with both clinical (LM) and engineering (RSD, NR) expertise, reducing the clinical-engineering disconnect.

The "Methods" section will outline the methods of the review. The "Patient and physiotherapist perceived limitations of exoskeletons" section will discuss the patient and physiotherapist perceived limitations of existing rigid exoskeletons based upon a review of the existing literature. The "Patient and physiotherapist perceived limitations of exoskeletons" section will also introduce potential solutions to these limitations. The "Discussion" section will summarise the main findings in relation to wider literature, and has a wider discussion on future assistive suits and identifies how soft-robotic technologies may provide solutions.

## Methods

Initially, the researchers individually scoped out the literature, to highlight areas of potential interest for review and used this initial exploration to define the aim, and subsequent scope, of the review. It was evident that there was limited literature on patient and physiotherapist perspectives on hard and soft exoskeletons (See the "Introduction" section). A decision was made to collectively explore both patient and physiotherapist perspectives so that there was sufficient literature to compile this review. A greater amount of discussion exists into rigid exoskeletons, and therefore, it was decided to focus the solutions on more novel soft exoskeletons. It should be noted that soft exoskeletons are preferred for users who retain some walking ability or only require partial assistance, whilst users needing significant body weight support require a rigid exoskeleton [43, 44]. Consequently, the findings of papers regarding rigid exoskeletons will contribute to the discussion of developing soft exoskeletons, but the reader should be mindful that the users may have different needs as we explore solutions for those who are further along their rehabilitation pathway and typically require less physical assistance.

In order to gain a greater insight into users’ perspectives, literature with qualitative and quantitative studies were included in the review. As exoskeletons have progressed significantly within recent years, it was preferable to have more recent papers. However, the Wollf *et al.* (2014) [34] paper was highly relevant to our review, and it was cited frequently in other literature regarding patient perspectives of exoskeletons. Consequently, papers were included if they were published after 2014. Following the guidance of Grant and Booth [45] regarding state-of-the-art-reviews, no formal quality appraisal was carried out. However, studies were only included if data collection methods were transparent so that the researchers could identify relevant study contexts (for example, the sample population) and whether correct research conduct was undertaken.

The review aimed to focus on finding solutions for exoskeleton issues that were user-led. Therefore, the review was led by a health researcher (LM) without an engineering background and without (at this time) active involvement in the technologies of soft exoskeletons. It was important that the engineers did not outline the perspectives of the users, as they would bring their own bias due to their knowledge of the technologies that they could develop.

LM independently searched and identified relevant literature using the databases MEDLINE, AHMED and CINHAL. Snowballing of papers was also carried out. LM collated the literature into a summary table and the selected literature was also discussed with MC and AT researchers with a physiotherapy and occupational therapy background, respectively. Themes were identified, which were recurring patterns regarding patient and physiotherapist perspective of exoskeletons across the papers. which included the themes which would become the framework for presentation of findings (Additional file 1: Table S1).

The themes were presented to the research team and then research team members with engineering expertise carried out further literature searches for engineering solutions. The scope of these searches were defined by the findings from LM’s reporting. This was to ensure that the solutions were based upon the user’s perspectives, and not any agenda of the engineering researchers. A narrative was then formed based upon the user’s needs, and how soft robotics may offer a solution. RSD and NR, engineering researchers with mechanical and soft robotic backgrounds, searched IEEE, SpringerLink, Scopus database for state-of-the-art technology that addressed assisting patients and therapists. They concluded findings, delivered engineering solutions to each theme and provided a guideline in developing key elements of assisting devices. Findings were shared with LM who compiled them in order to create a narrative for the review.

## Patient and physiotherapist perceived limitations of exoskeletons

The findings’ framework is based upon six themes: safety, one size fits all, ease of device use, weight and placement of device, cost of device, and appearance. Themes did not change from LM’s consultation of MC and AT, however, the presentation of themes were ordered to reflect the weighting of the literature for the themes. Themes are presented below with their relevant findings. Five papers were included that evaluated patient and/or physiotherapist perspectives of exoskeletons.

### Safety

Across the sources, safety concerns regarding use of rigid exoskeletons can be divided into: primary harm incidents, secondary harm, and concerns of infection control.

Several concerns were expressed regarding primary harms that could be caused by a lack of device sensitivity and sophistication. In one qualitative study, physiotherapy student views on the H2 rigid exoskeleton were explored; they had not used the device but had only seen a participant don/doff the device or had the process described to them (two out of three focus groups were online) and viewed videos of people walking with assistance from the device [18]. The students felt that the device may force a patient to go past their joint range of movement, causing injury [18]. Similarly, a physiotherapist in Vaughan *et al.*’s study [25] (who had actually used the device for one year) expressed concerns for how the device would respond to atypical muscle tone, providing an imagined example of a patient with hyper-reflexia and the device misinterpreting this as sitting. Physiotherapists in Read *et al.*’s study [31] had undertaken training with the Ekso rigid exoskeleton and used it as part of their interventions. The physiotherapists highlighted that this device could cause a larger degree of spasticity, due to patient anxiety for use of the equipment. Thus, it has been perceived by physiotherapists that the device could not only cause injury in its response to muscle tone, but it could exacerbate the problem. It should be noted that although these concerns were not based on actual experiences, nonetheless, they remained present even after training and use of the devices for several months (training spring/summer 2017 and data collection July 2017).

Primary concerns also included the device being inappropriate for particular patient groups. Physiotherapists were apprehensive that, for patients with limited core strength, the device may impact their balance and cause injury, with a physiotherapist referring to it as ‘throwing them through the motions’ [25] (p0.11). Concerns regarding falls were shared by several users of wheelchairs [34]. Consequently, a physiotherapist (with no experience of using the device) stated that they would only feel comfortable using the device if they maintained close proximity when mobilising patients [25]. There were reservations for using the device with patients with cognitive and communication deficits; however, discussion was limited [18, 25].

A secondary safety concern, with expression limited to one physiotherapy student, but with particular relevance in light of the COVID-19 pandemic, was the ease of cleaning the device [18]. Predominantly, secondary safety concerns were in relation to creating one device to fit all.

Compliance as a physical property is one of the most important required features in developed exoskeletons, facilitating adaptability, comfort and safe interaction with human body [46]. Series Elastic Actuators (SEAs) and Variable-stiffness Actuators (VSAs) are commonly used in conventional rigid spring-based exoskeletons because of their ability to change their stiffness [40]. Despite comprising rigid elements, SEAs and VSAs are considered compliant actuators with the capabilities of transmitting high forces and providing smooth assistance and avoiding restriction of natural body motions and injury. However, they have certain disadvantages such as mechanical friction and hysteresis (actuation delay). Often, the empirical behaviour such as the device’s practical stiffness does not match with prediction [40]; the fitting of the rigid exoskeletons determines the expected rotation axis of the joints and, therefore, it dictates the constraints and an undesired range of movement applied to the person. In addition, traditional rigid exoskeletons exploit self-adjustable body attachments to compensate joint misalignment [47]. For example, a rigid full-DoF hip exoskeleton containing rotational hinges and perpendicular sliders has been shown to passively adapt to align the exoskeleton’s components with the user’s specific body biometrics, reducing undesired interaction forces [48]. Similarly, iT-Knee is a self-aligning rigid knee exoskeleton, delivering autonomous adaptability, while assisting lower limbs, and pure knee assistance decoupled from other joint movements [49]. However, inclusion of these joint-aligning mechanisms increases the complexity, weight and rigidity of exoskeletons.

The decrease in rigid components can be observed throughout the history of developments in exoskeletons using a variety of soft robotics technology to pursue totally soft exoskeletons, which is safer, more comfortable and friendly to users (Additional file 1: Fig. S1). Pneumatic Artificial Muscles (PAMs) are common soft actuators that change shape and exert forces when pressurised by air [50, 51]. PAMs have been used for rehabilitating soft exoskeletons over several decades [52–66]. Cable-driven soft exoskeletons consist of minor rigid components (e.g. motors, gears and cables) which use textile or soft attachments to deliver direct force transmission and comfort to a user’s body during assistance. These actuators are predominantly off-board (not attached to the suit, but tethered to the suit through cables), reducing the suit weight loaded on a user’s body and making them suitable for use with a therapist who is able to assist carrying the actuator [43, 67–71]. Moreover, more recent PVC gel electroactive polymer actuators have been used to build soft exoskeletons [72]. When assisting the human body, it can decrease the activity and energy spent by the targeted muscles [73]. However, there may be safety concerns due to the high voltage supply required.

### One size fits all

Although rigid exoskeletons are designed with the intention to facilitate motor learning of a typical gait pattern, physiotherapists felt that the device may cause unnatural movement. A pre-fixed gait pattern in the sagittal plane was perceived as imposing a gait pattern on a patient that did not correlate with real life walking [18]. Moreover, it was highlighted that a one-size-fits-all device had unnatural hip/pelvic alignment and knee alignment, and subsequently impacted their base of support and caused new compensatory movements [25]. This was the experiences of several stroke survivors who used a rigid exoskeleton and stated that the device ‘felt unnatural’ and made it harder to transfer their weight between legs [25] (p0.7). Furthermore, there were concerns that providing too much assistance to an individual could cause passivity or increased dependence. A physiotherapy student voiced concerns that patients may be unaware of the feedback they are receiving from the rigid exoskeleton, which can lead to device dependence [18]. This was the experience of a stroke survivor; they felt the rigid exoskeleton’s pre-programmed gait pattern was walking for them, rather than providing the assistance they required [25].

Consequently, physiotherapists requested an exoskeleton that they could tailor to the needs of each specific patient. For instance, they wanted to adjust joint angles, length of the femur and the swing pattern [25]. Physiotherapists highlighted the issue of time consumed from altering an exoskeleton for each patient if it were in a rehabilitation facility clinic, and suggested that the device have a function to retrieve patient specific settings [25]. Rather than a one-size-fits-all device, both physiotherapists and several stroke survivors felt that a range of exoskeletons may be required, to meet the differing needs of early and later stages of rehabilitation, in both acute care and community rehabilitation [18, 25].

There were concerns that it might not be possible to have an exoskeleton that is suitable for all [31]. Pragmatically, physiotherapists expressed issues in having one device that can physically fit every patient, and they felt skills may be required to fit the device so that it was comfortable for the patient for an entire session [31]. In a survey exploring how users of wheelchairs perceive exoskeletons, some were concerned that the device would not be suitable for their impairment, with examples provided include: hemiplegia, quadriplegia, low bone density, contractures, lack of arm/hand use, poor balance, amputation, obesity, muscular dystrophy and asymmetrical lower extremities [34]. However, as this was an open-ended survey question, there was limited depth into *why* patients perceived these impairments as preventing their use of exoskeletons.

From the engineering perspective, the goal of soft assistive devices is to generate predefined trajectories that train patients and recover their body motions, while simultaneously adapting to each patient’s specific needs. Therefore, rehabilitative devices must determine the assistive conditions, estimating the amount of required assistance, and timing of activation and withdrawal of assistance [74]. However, these parameters vary significantly between patients, causing time-consuming adjustment.

A sophisticated control algorithm, called human-in-the-loop optimisation was developed for cable-driven exoskeletons to solve these issues. The algorithm is able to adapt its assistance strategy based on the individual’s walking performance [69, 70]. Although starting with a standard walk-assisting force profile, this algorithm enables the exoskeletons to rapidly adjust the assisting force and provide precise actuation to match the cyclic walking motions, improving and maintaining optimal walking performance at all time [69, 70].

Flexibility and adaptability, which may be offered by novel textiles, are the key solution to the problems of rigidity and low adaptability with existing exoskeletons (See the “Safety” section). Flexible textile materials, such as soft braces, straps and garments, are currently the most effective solution for the compliant connection between an exoskeleton and the user’s body. They are used to transfer assisting forces and hold the suit in an appropriate configuration, providing compatibility with natural motions and safety [40, 75]. Moreover, semi-soft exoskeletons, containing rigid components and soft attachments, were designed to include adaptability by changing the length of their rigid linkages to fit various body sizes. However, this component-adjustment process consumes considerable time for each patient. Alternatively, variable-stiffness materials may be integrated into body attachments. For example, a 3D-printed variable-stiffness structure stimulated by heat and electricity may be incorporated into adaptive body attachments [76].

### Ease of device use

The ease by which an exoskeleton can be used was a common theme across several studies [18, 25, 31, 34, 35]. Wollf *et al.* [34] highlighted that, out of 17 criteria for importance of exoskeleton design features, ease of device use was number 4 for users of wheelchairs. Bortole *et al.* [35] highlighted that, for their sample of stroke patients, ease of exoskeleton use was ranked as 7.2 on a Likert scale, with 10 being ‘extremely easy to use’; one patient expressed that ‘wearing it is fast and simple’ (p0.11). Time was a common rationale for wanting the exoskeleton to be easy to use, which was frequently related to donning and doffing the device [25, 31, 34]. One stroke survivor stated that it took 30-40 minutes to fit the device which they felt was ‘lengthy’ (p0.5), while another stroke survivor did not want half their therapy time being absorbed in this way [25] Read* et al.* [31] stated that sessions took 60 minutes using the Ekso rigid exoskeleton and 90-120 minutes for the initial assessment, and physiotherapists highlighted the time-consuming nature of the device.

Time management was also discussed in relation to the training required for physiotherapists to use the device. Physiotherapists felt this training had to be multi-faceted to address the complexities of the device, which included: device-specific technical know-how; maintaining patient safety; the necessity of working within time constraints; deciding the appropriateness of the technology in a given situation; accurately acquiring the measurements required for proper fit; a need to check and recheck that all is operating as intended; and an understanding of patient needs [31]. The physiotherapists perceived the training sessions as ‘challenging’, and it was stated that they felt the skills had to be maintained (p0.5). Physiotherapists in Vaughan-Graham *et al.* [25] perceived it as essential for ongoing support and training, while physiotherapy students wanted a contactable technical expert in case of exoskeleton issues [18]. Physiotherapy students felt that training may be a deterrent for the exoskeleton’s use, particularly for less experienced therapists who find time management more challenging [18].

There were also ease of use factors that were specific to certain patient groups. This included the ability to use exoskeletons without crutches, which was ranked as 3.71 out of 5 on the Likert scale in terms of importance of design feature (ranked 14 out of 17) [34]. This was expressed as important to participants with hemiplegia, Muscular Dystrophy and users of crutches who wanted to be able to have free use of their arms while carrying out activities such as cooking [34]. Physiotherapists also expressed concerns for ease of use for patients with cognitive, perceptual, and communication impairments, and the potential for harm while using the device [18, 25].

In terms of the design considerations for engineers, it is essential that the device is easy to put on and take off. A soft trouser, which integrates soft robotic actuators, (rather than a mechanical assembly that constrains and anchors certain areas in the body) is easier to wear and reduces unproductive time during therapy. The challenge remains for the soft structure to effectively interface with the body and to provide sufficient torque/force and tension/compression to deliver mechanical assistance as required [46]. A lightweight, portable, active undressing trouser [77] is a good example of a compliant exoskeleton integrating a soft pneumatic adaptive belt inside a regular trouser. It can expand and loosen due to the pressure input supplied by a compressed gas cartridge, allowing ease of donning and doffing. As mentioned previously (See the “One size fits all” section), the autonomous control-optimising algorithm can also vitally decrease time and effort therapists spent to manually adjust the exoskeleton setting for each patient [70].

### Weight and placement of device

Studies commonly referenced weight, size and placement of exoskeletons. In the Vaughan-Graham et al. study [25], both stroke surivors and physiotherapists were concerned about the weight of the rigid exoskeleton; in particular, therapists felt the device’s pelvic placement could result in the patient’s centre of mass being shifted backwards, disturbing their gait pattern. Wollf et al. [34] survey demonstrated that, for users of wheelchairs, portability of the device was 4.09 out of 5 in importance of exoskeleton features (ranked 10 out of 17 features). In the same study, battery life of the device was ranked number 6 - a consideration that was shared by physiotherapists - suggesting that a potential trade-off exists between operating time and device weight/portability [25, 34]. A further design consideration highlighted was the potential for skin breakdown due to pressure sores; however, this response was limited to one patient [34].

Reducing the weight of exoskeletons has been a key focus for engineers designing rehabilitative and assistive devices. Existing rigid portable exoskeletons aim to restore mobility of disabled patients, examples include Exo-H2, Indego, ReWalk and EksoNR, have masses between 11 and 25 kg [78]. They can support and move patients with mass over 100 kg. In contrast, compliant exoskeletons using off-board tethered cable-driven actuators and light textile attachments to transmit assisting force to a user’s body have significantly lower masses of around 0.9 kg (weight of suit alone), dramatically reducing parasitic or undesirable loads on the body [43, 68–70]. Although extremely lightweight compliant assistance devices have been shown to improve walking, their operation has been limited to treadmill training because of difficulty in carrying off-board, heavy actuation units and energy source. Myosuit [71] and XoSoft [79] are examples of portable cable-driven exoskeletons including on-board actuators with entire masses of 4.1 and 4.6 kg, respectively, significantly lighter than a contrasting cable-driven exosuit (12.15 kg) [67]. The mass of pneumatic-driven exoskeletons can be even lower (less than 0.16 kg); however, this excludes the weight of the heavy pumps and compressors required for the air supply [63, 66].

Placement of body attachments is another critical part of exoskeletons. Key anchors, such as the shoulder, the iliac crest of the hip and the plantar surface of the feet, are defined as effective body locations onto which to attach assistive devices [57]. These specific areas have the thinnest skin above the bone compared to other surrounding areas of the body, which can prevent misalignment, pressure sore, skin damages and muscle injuries while transmitting assisting forces to the skeleton.

### Cost of device

Cost of exoskeletons was a consideration shared by both physiotherapists and patients [18, 25, 34]. The survey of wheelchair users highlighted that the exoskeleton cost was second in importance only to design features (2/17), with the open-ended responses demonstrating several patient concerns of affordability [34]. Physiotherapists likewise discussed the concerns for the cost of any devices to patients [25]. Furthermore, physiotherapists and physiotherapy students perceived the difficulty in funding this across a facility budget, and a private clinic, respectively [18, 25]. A physiotherapist felt that, if the device were to be shared across entire programs, there could be added complications regarding bookings, and another physiotherapist remarked that there had to be a significant benefit in order to justify the cost [25]. It was perceived that the cost of exoskeletons could hinder clinical uptake [18, 25].

To the authors’ knowledge, there is no literature on cost evaluation of exoskeletons or use of cost reducing materials. However, the soft exoskeletons can generally separate into two major parts: a suit with integrated soft actuators, and a power-supplying unit. Presently, there is an increasing trend to use more fabrics to create soft exoskeletons [80]. These soft devices are low cost and affordable, and they have the additional benefit of minimising the required power-supply unit, thus increasing its portability.

### Appearance

Appearance of exoskeletons was a patient consideration; however, it was ranked as the least important device criteria for users of wheelchairs (3.23 mean importance out of 5) [25, 34]. There appeared to be variation in expectations of the exoskeleton appearance; one participant remarked that they did not want to look like a robot and the device needed to be wearable under clothes, while another participant expressed accepting the limitations of the device appearance in order to improve their mobility. The minimal level of importance of device appearance in the Wolff et al. [34] survey only provides an average of mean importance and it may not reflect the strength and variety of opinions of patients.

Existing soft exoskeletons have been designed with aesthetics and discretion as important considerations, while maintaining high body-assisting performance [46]. Textile materials and garments have been used as the main components of the soft exoskeletons to safely interact with a user’s body, delivering assistance in a discreet manner. One excellent example is integrating soft actuators inside a normal trouser for undressing assistance [77].

## Discussion

This state-of-the-art review has highlighted a range of patient and physiotherapist perceived concerns and design considerations regarding rigid and soft exoskeletons. Patient and physiotherapists had reservations regarding rigid exoskeletons’ safety, such as falls, despite not having an experience that would warrant this or having seen wider evidence that demonstrates that falls were only present in testing of early exoskeleton prototypes [81]. The significance placed upon falling is unsurprising; falls have widespread physical and psychological impact on individuals and a survey of wheelchair users demonstrated that minimising the risk of falling was their top priority when evaluating exoskeleton function [34]. Other concerns shared by the users of rigid exoskeletons were that the device may force patients into unnatural movement patterns, or even erroneously detecting and forcing movements, for instance, interpreting the patient as trying to sit when they are in fact walking. This paper then explored the use of soft technologies with greater sophistication, which are more sensitive to the users’ movements and are thus able to more precisely apply forces as required.

From an engineering perspective, the first challenge is developing new soft actuators that are lightweight, compliant and sufficiently powerful enough to deliver smooth and safe assistance for natural mobility [82, 83]. They must be capable of varying their stiffness and deforming when exposed to external forces generated by patients, reducing unexpected harms. Furthermore, they must be suitable to fit inside a garment-like suit that is wearable and comfortable. The level of stiffness is directly related to the amount of force that can be transmitted to the human body; therefore, the balance between stiffness and assistive effectiveness must be considered. For example, compliant actuators can deliver safety and comfort together with portability due to their low weight, but may have decreased bandwidth or peak force when compared to conventional heavier rigid actuation technologies such as motors and gears [46]. Additionally, actuation speed must be considered when delivering assistance that merges seamlessly with natural body movements, since asynchronous actuation can result in increasing muscle power consumption and fatigue of users and negative changes in mobility patterns.

Both physiotherapists and patients were concerned that if there were only one exoskeleton device that was made to fit all patients, it may result in unnatural movements [18, 25], which was the experience of users in the recent Vaughan et al. study, where there was unnatural hip/pelvic alignment [25]. A longitudinal study of healthy participants demonstrated the individual nature of gait characteristics, and concluded that assessment and therapy must therefore take into account the patient’s unique differences [84]. This review highlighted that physiotherapists wanted a device that they could tailor to an individual [25]. However, they did note that altering a device between patients may be time-consuming [25], and they were concerned that it may not be possible to have a device that can physically fit all patients [31]. It was important for the device to be easy to use, both in terms of donning/doffing the device and training. However, rigid exoskeletons were not easy to use, with one study citing 30-40 minutes simply to fit the device [25]. As previously highlighted, typical therapy time for stroke survivors is already below the recommended 45 minutes per day [10]. A systematic review exploring the clinical applications of the HAL exoskeleton [28] found that, of the three papers that reported the breakdown of the time using the devices, all the sessions took 90 minutes, with one study reporting up to 60 minutes effective time, while the other two reported only 20-30 minute effective therapy time [85, 86]. Devices can be initially time-consuming; National Institute for Health and Care Excellence (NICE) [87] states that training physiotherapists to use an Ekso exoskeleton takes one week and the therapist must initially only use the device under supervision of a physiotherapist who is familiar with the Ekso. It is evident that future devices must be more efficient in relation to the required training and time taken to use the device.

We perceive that the future exoskeletons will become totally soft and naturally integrate with normal clothing. Their actuators and body attachments will be combined to create multifunctional assistive clothing with capabilities of stiffness variability and morphology deformation on every areas of the suit. For example, at no assistance, the entire suit turns soft, improving ease of donning and doffing. Moreover, it is perceived to be able to harvest energy gained from passive shape deformation and heat loss released from the body surface during daily activity. Also, when activated, certain areas of the suit can be controlled to become stiffer to support loads on lower limbs or to prevent undesired movements and joint misalignment. In contrast, other areas can actively vary their stiffness to deform, e.g. contract, elongate, bend and twist, and enable actuators to effectively transmit forces to assist required body movements and also prevent joint misalignment. In addition, active morphology deformation can enable suit adaptability to specific users and self-fitting for comfortable usage by controlling shrinking and loosening of the suit to fit the user’s body. A variety of developed textile actuators was classified depending on their purpose and reviewed in [75].

Physiotherapists and patients all highlighted the importance of having a device that was lightweight, to prevent the user’s gait pattern being altered [25] and to allow it to be portable [34]. However, a long battery life was also desired, suggesting the need for a balance between a lightweight portable device that can operate for a short period and a heavier device that has longevity of use [34, 88]. As therapy time is limited to 35 minutes for stroke survivors [10], this would be the minimal time accepted, however, if the device is to be used by multiple patients in a day, greater device battery capacity or a short recharge time may be required. The requirements for a lighter assistive device are soft and efficient actuators (high torque-power and torque-weight ratio) and power sources that have short recharge time. However, both these requirements urge development in the material science. Within the current technology, wireless charging during users’ session can be adopted to optimise standalone charging time.

The cost of the device was important to both patients and physiotherapists, in both terms of cost for the patient to buy for personal use, and also the purchasing by clinics [18, 25, 31]. Physiotherapists would only want to purchase a device if the device benefits clearly outweighed the cost [25]. In the UK, there are several bodies involved in making budget decisions, passing from the Department of Health and Social Care, to National Health Service then to Integrated Care systems (previously Core Clinical Commissioning Groups) [89, 90]. It can be expected that approval from commissioners for high-cost services or products, such as soft exoskeletons, will be challenging. An economic evaluation explored the cost-effectiveness of rigid exoskeletons in improving quality of life and preventing secondary hip fractures in an imagined population of people with dementia or cardiovascular diseases [91]. The multiple scenarios demonstrated that a significant improvement in reducing hip fractures was not essential, however it was essential to improve quality of life in order to justify the cost of the exoskeleton (with the cost under £17,500) [91]. In 2017, Ekso was provided to one NHS Trust in a package costing £98,000 (excluding VAT), which included the Ekso GT robotic exoskeleton with the SmartAssist software, training for up to four physiotherapists, a two?year warranty, supporting equipment [92]. This cost is significantly greater than the value outlined in the cost-benefit analysis, and it is therefore evident that future exoskeletons must have greater affordability.

Although the appearance of rigid exoskeletons was commonly discussed by patients, their views varied, with some feeling appearance was irrelevant if the device allowed them to live their life, and others not wanting to feel stigmatised by a visible device [34]. This demonstrates the individuality of patient needs when designing future exoskeletons.

To build the next generation rehabilitative device, we propose that developments are required in i) building efficient soft robotic actuators, ii) fabricating comfortable body attachments, iii) improving sensor technology and iv) developing robust adaptive control strategies.

### Soft robotics actuators

Similar to the key metric performance of human skeletal muscles, soft fluidic and electrically-driven actuators have high potential to become future smart artificial muscles due to their high stress, strain and bandwidth, addressing the requirements to create the future soft exoskeletons [93].

Soft fluidic actuators have backdrivability (behaving like a spring), low cost and high specific force and power, despite low efficiency around 30% because of losses in the fluidic-to-mechanical energy conversion process [40, 93]. They can be divided into hydraulic and pneumatic actuators. Soft pneumatic actuators have lower bandwidth than hydraulic actuators due to gas compression, but are significantly lighter, making them more suitable to create soft exoskeletons (see Additional file 1: Fig. S2 for the vision for future assist devices). Many prototype soft lightweight exoskeletons were built using PAMs [53], for example, McKibben muscles [51, 94], straight-fibre muscles [95–98], Pouch Motor actuators [99] and pleated PAMs [100] including Bubble Artificial Muscles [64, 65]. However, one drawback of pneumatic artificial muscles is the requirement for large, heavy, noisy pumps or compressors, which limits their portability for ambulatory applications.

Soft electrically-driven actuators are another potential candidate for future soft exoskeletons due to their high actuation performance, fast and quiet operation, and high efficiency [46, 93]. For example, dielectric elastomer actuators (DEA) and ionic polymer-metal composites (IPMC) are active polymers which deform when electrically charged. DEAs can deliver high strain, bandwidth and efficiency, and are widely used in robotic applications [101–103]. However, the major disadvantage of DEAs are the requirement for high voltage actuation (typically in the order of thousands of volts). Alternatively, low voltage actuation is achieved in IPMC (in the order of a few volts) but at the cost of lower stress and power density [93]. Dielectric fluid electrostatic actuators have been developed as a new soft robotics actuation technology. Examples include dielectrophoretic liquid zipping actuator [104–106], hydraulically amplified self-healing electrostatic (HASEL) actuators [107–109], soft fluidic pumps, such as a stretc.hable pump [110] and an electro-pneumatic pump [111, 112]. The recent stretc.hable pump and electro-pneumatic pump demonstrated important capability to integrate with soft fluidic actuators, e.g. hydraulic McKibben muscles [113] and pneumatic Bubble Artificial Muscles, respectively. Together with the development of lightweight battery technologies and on-board controllers, these soft electrostatic actuators promise a new generation of entirely soft, lightweight, flexible and portable exoskeletons. Although their stress-strain performances are lower than human muscle requirement, they combine the benefits and address the drawbacks of both fluidic and electrically-driven actuators.

Auxetic or transformable structures has been explored to develop ankle and knee braces that are lightweight and comfortable, similar to a textile or garment [114]. Exploiting the advancement of additive manufacturing and 3D printing, the transformable structure can be actuated using micro pneumatic actuators or by pulling threads. There are numerous auxetic structures available, and their potential has not been fully investigated in actuation. Many of the transformable structures can bend, twist and contract, and their characteristics can be altered depending on the way they are placed, such as unit type (e.g. block size, number of edges, number of hinges/constraints or layers) and connection type (e.g. series or parallel). These structures can be easily incorporated within the garment and their ability to transform in shape can be used to move joints or alter the local stiffness of parts of a suit. A combination of different auxetic families in a garment could be helpful to obtain desired strains in different activities such as sit-to-stand or walking. These make active auxetic structures another potential solution to develop future soft exoskeletons. Additional file 1: Fig. S1 demonstrates the perceptual concept of the future assist device, illustrating soft artificial muscles, sensors and a power supplying unit.

### Body attachments

The conditions of body attachment, such as size, shape and attachment point, play a crucial role in patient comfort that form an essential focus of evaluation for the performance of soft wearable exoskeletons. As previously discussed, the safe placement for attachments of the soft exoskeletons on the user’s body is a shoulder, hip and feet [57]. In addition, a guideline for designing aesthetic and inconspicuous exoskeletons is provided in Veale et al. [46]. That is, the loads from the suit mounted on trunk and each foot are recommended to be less than 15% and 1.25% compared to the user’s body weight, respectively. The thickness of the suit along lower limbs should be less than 30 mm. The power source can be located on the user’s back with the total volume limited to 0.023 m$^{3}$.

Although not considered by the patients and physiotherapists, we perceive that breathability is an important feature of body attachment and can significantly enhance comfort while wearing the soft exoskelton. This can be achieved by temperature and humidity exchanges on the skin surface. For example, the suit can possess self-deformation, which is sensitive to skin surface temperature and humidity and autonomously deforms its structure to inhale/exhale surrounding air for cooling/warming of the suit [115].

### Sensing system and mobility simulation

Typically, exoskeletons require a sensing system to be highly accurate, fast, and robust to disturbances. The sensing system is accountable for three fundamental features: (1) measuring force generation, (2) monitoring body motions, and (3) evaluating assistance efficiency. First, the soft exoskeletons require sensors to measure their actuators’ outputs, which are then fed back to their controller to deliver accurate assisting force and strain. Sensors are also used to prevent undesired damage, for example, stopping actuation when high forces and out-of-range motions are detected.

Second, IMU (inertial measurement unit) sensors are commonly used to track and simulate body movements by their attachment across body joints. With the development in a real-time data-analysing system, IMUs were able to precisely predict walking and sit-to-stand activities [116, 117]. Alternatively, soft flat sensors, such as DEA stretc.h sensors [118] and a multi-bend/shape sensor [119], which can contract, extend, bend and twist, may be integrated as one layer of the soft exoskeleton. These soft sensors can be used to measure and simulate the entire 3D configuration of the suit in order to predict mobility activities and to detect incorrect mobility symptoms or patterns. Soft force sensors can be intergrated in the the soft exoskeleton to estimate the pressure distribution on the user’s skin and thereby to prevent skin damage, especially around the attachments, and adjusting the assisting strategy or altering the exoskeleon morphology for more user comfort and safety.

We envision that all of these functional sensors need to be soft and aesthetically embedded within soft exoskeletons to comprehensively monitor the user’s body. The future of assistive soft robotic clothing may include multiple layers that contain actuating, sensing and control units, operating synchronously to facilitate a person’s improved mobility.

As a result of the body motion information acquired from suit sensors, a virtual mobility simulation can be simultaneously created. Additionally, an open source platform that can capture the motion from the suit with plug-and-play capabilities may facilitate rapid analysis of patient mobility patterns. The integration of the above technologies with an online data communication and service has the potential to track mobility improvements and enable remote patient consultations with physiotherapists.

### Control algorithm

Exoskeletons must always generate the appropriate amount of assisting forces at the right time for effective assistance without negatively impacting a user’s mobility. As previously shown, human-in-the-loop optimisation was developed as an advanced control algorithm which can automatically adapt the assisting strategy based on the user’s mobility, consistently delivering effective, harmless and optimal assistance [69, 70]. Although based on the same predetermined assisting profile, this algorithm can rapidly adjust itself to suit the mobility needs of a variety of different patients, conserving therapy time. In order to deliver such sophistication, future exoskeletons require a soft, high-speed, micro-scale, computing controllers that can be unobtrusively distributed across the suit and which communciate and integrate to deliver low-level mechanical assistance to deliver a high-level mobility goal.

### Beyond soft-robotic solutions: future research

Implementation will only be successful if devices are themselves effective and that this can be proven in clinical and usability trials. A recent Cochrane review [120] of 62 trials (totalling 2400 participants) was carried out to determine whether electromechanical-and-robot-assisted gait training versus normal care after stroke improved walking. It concluded that stroke survivors who received electromechanical-and-robot-assisted gait training alongside physiotherapy were more likely to achieve independent walking than those who carried out gait training without these devices. However, the study concluded that questions remained regarding the most effective frequency and duration of the training and which design characteristics were important for bringing about the improvement. This was in part due to differences in device design, for instance, some devices having FES. Further, the review concluded that the variation in time since stroke needs to be considered in future testing as the training may not benefit those who were ambulatory at the beginning of the intervention. Consequently, it is necessary to understand specifically *what* about devices make them ‘effective’ and for *who*, and what outcome measures class this ‘effectiveness’.

However, measuring effectiveness is a problem that is widespread across the robotics field. A systematic review [121] explored the effectiveness of platform-based robotic rehabilitative devices (devices which solely improve ankle performance) for use with people with musculoskeletal or neurological impairment. They could only conclude on the effectiveness of two devices due to the availability of evidence. However, they found both devices to be effective in improving ankle range of movement and stability. They proposed that, to reach further conclusions for these devices, there must be future work into creating universally accepted evaluation criteria which are able to standardise the devices’ outcome evaluations. But this is not without its challenges. Flynn et al. [122] explored the sustainability of upper limb robotic therapy for stroke survivors in an inpatient rehabilitation setting. They highlighted that robotic devices can create very subtle improvements or change in quality and degree of movement, which may be missed by less sensitive commonly used outcome measures. They underlined the need for an understanding of the clinical reasoning that underpins the prescription of robotics for upper-limb therapy by physiotherapists, as well as the most effective time to incorporate it into rehabilitation. This study is based upon upper-limb therapy, however, it is still relevant to the development of lower-limb therapy. Evidence demonstrates the need for greater guidelines for robotics in rehabilitation in general, which may encourage a ‘cultural shift’ for robotics acceptance [123].

Fundamentally, there must be an understanding of *how* therapists use devices and *why* therapists use devices in the way they do; this is essential to not only design robotic assistive devices that meet therapists’ needs, but also to create guidelines that can facilitate successful implementation of such devices. Notably, the need to understand the therapist’s clinical reasoning and the devices outcomes are interconnected. As Vaughn et al. [25] study exploring exoskeleton use in stroke rehabilitation concluded, unless client selection criteria and goals of device (both clinically reasoned) are established, there is a risk of exoskeletons lacking statistically significant effect. Future research should use interviews and focus groups with therapists not only to explore their clinical reasoning when using devices, but also to understand how devices might be tested meaningfully for their purpose, in a standardised way.

This review identified that physiotherapists felt that training professionals in exoskeleton use had to be multi-faceted, and that the training was often negatively perceived to be draining. Stephenson and Stephens [123] explored physiotherapists’ experiences of robotic therapy in upper limb rehabilitation within a stroke rehabilitation centre. Although initial starting up times for robotic devices may be greater than conventional methods, it was perceived that the devices could free up more experienced therapists’ time. However, they acknowledged that, without a greater evidence base, it was challenging to set the parameters for training. This reinforces our conclusion for the need to create clear clinical guidelines for device usage (clear device functions, recommended usage time and appropriate patient groups). Additionally, they concluded that close partnerships between the technical device manufacturers and the professionals using the devices were essential for successful training of staff, problem-solving and maintenance of equipment [123]. This further highlights the requirement for inter-disciplinary collaboration for robotic device uptake.

## Conclusion

We present state-of-the-art technologies and examples of current rigid and soft exoskeletons which have been designed to address the needs highlighted by physiotherapists and patient users. This review has demonstrated a range of issues with current rigid exoskeletons, with limitations related to device safety, one-size-fits-all feature, ease of use, weight, placement, cost, and appearance. We provide a concept of future exoskeletons and soft-robotics solutions classified into four key factors: soft robotic actuators, body attachments, sensing system and control algorithm. The solutions proposed are extensive and the considerations when designing future soft exoskeleton devices are complex.

Soft pneumatic-driven and electrically-driven actuators are perceived as having the highest potential for future soft technology exoskeletons thanks to their high flexibility, speed of response and portability, and stress and strain performance that matches or exceeds human muscles. Applying compliance to all components of exoskeletons can increase users’ comfort and help prevent harm if assistance is misaligned or unsafe movements occur. Variable-stiffness capability can stiffen certain areas of the soft exoskeletons to maintain and deliver sufficient force transmission through the device to effectively assist lower limbs. This property also provides the devices with adaptability to deform themselves to fit to various specific users’ requirements, increasing ease in donning/doffing. The assistive device will use advanced sensors to detect user intention and an adaptive control algorithm will learn users’ patterns of movement over time to better respond when needed. Future devices must have these features in order to be fit for purpose in time-restricted therapy sessions, which was a key challenge of current exoskeletons. Textiles are recommended as the basis for developing future soft robotic exoskeletons, enabling them to resemble normal clothing, and enhancing aesthetics and user acceptance.

We hope that the findings of this work may facilitate engineers in designing softer, more comfortable and more effective exoskeletons that meet the multi-faceted needs of their patient and physiotherapy users. In light of the findings of this review, it is strongly recommended that any assistive device development is supported by continuous user involvement and consultations.

## Supplementary Information

**Additional file 1. **Additional figures and tables.
