# Supplementary material for: The-state-of-the-art of soft robotics to assist mobility: a review of physiotherapist and patient identified limitations of current lower-limb exoskeletons and the potential soft-robotic solutions
Source: J Neuroeng Rehabil. 2023 Jan 30;20:18. doi: 10.1186/s12984-022-01122-3 (PMC9885398; doi:10.1186/s12984-022-01122-3)
Supplement: Supplementary file 1 — Additional file 1. Additional figures and tables. [file 12984_2022_1122_MOESM1_ESM.zip › 12984_2022_1122_MOESM1_ESM/Figure S1.pdf]

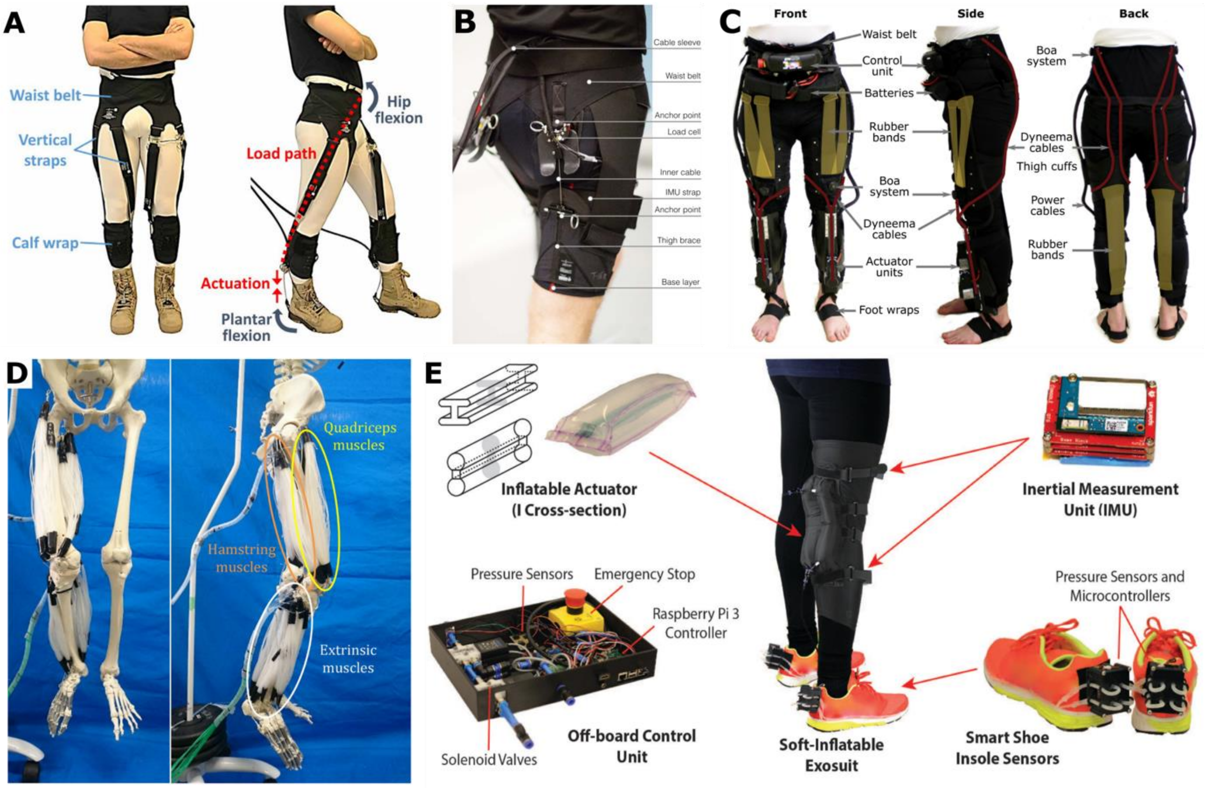

Figure S1 – Development of soft exoskeletons: (a-c) cable-driven exosuits and (d-e) pneumatic exoskeletons. (a) A tethered ankle-assisting exosuit [64] and (b) a tethered hip-assisting exosuit [67] for walking assistance. (c) An untethered Myosuit assisting hip and knee extension/flexion for sit-to-stand motions [68]. (d) Multifilament muscles consisting of several thin McKibben pneumatic muscles for a musculoskeletal lower-limb robot, mimicking human body motion [57]. (e) A soft-inflatable exosuit for knee extending rehabilitation during walking [59].
