# Supplementary material for: The-state-of-the-art of soft robotics to assist mobility: a review of physiotherapist and patient identified limitations of current lower-limb exoskeletons and the potential soft-robotic solutions
Source: J Neuroeng Rehabil. 2023 Jan 30;20:18. doi: 10.1186/s12984-022-01122-3 (PMC9885398; doi:10.1186/s12984-022-01122-3)
Supplement: Supplementary file 1 — Additional file 1. Additional figures and tables. [file 12984_2022_1122_MOESM1_ESM.zip › 12984_2022_1122_MOESM1_ESM/Figure S2.pdf]

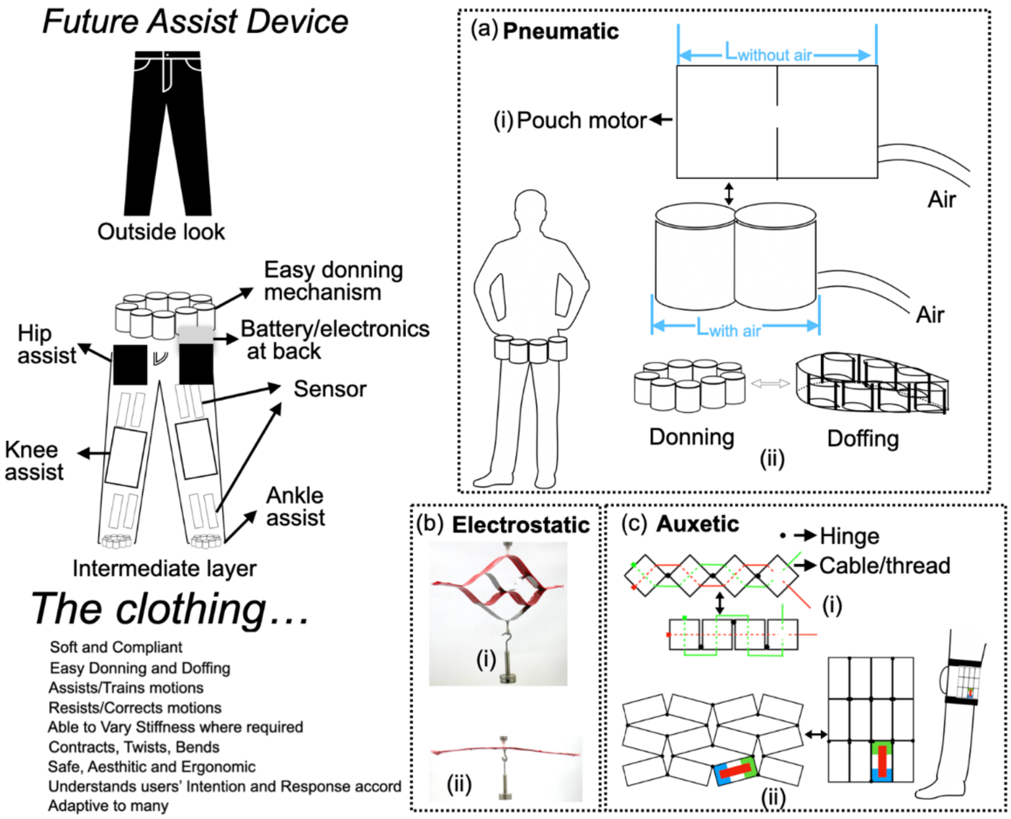

Figure S2 – The vision for Future Assist Devices as soft and compliant garments that are easily wearable. Pneumatic, Electrostatic and Auxetic examples that have potential to be integrated with the garments for the next generation assist devices; a)-(i) describes the principal of Pouch actuator [96], when the cylindrical chambers are inflated, the length of the actuator decreases and a)-(ii) this concept can be implemented in donning and doffing, b)-(i) and b)-(ii) Electro-ribbon actuator presented in [101], and c)-(i) auxetic structure that bends, elongate, twists, and c)-(ii) transformation of an auxetic structure.
