# Supplementary material for: The-state-of-the-art of soft robotics to assist mobility: a review of physiotherapist and patient identified limitations of current lower-limb exoskeletons and the potential soft-robotic solutions
Source: J Neuroeng Rehabil. 2023 Jan 30;20:18. doi: 10.1186/s12984-022-01122-3 (PMC9885398; doi:10.1186/s12984-022-01122-3)
Supplement: Supplementary file 1 — Additional file 1. Additional figures and tables. [file 12984_2022_1122_MOESM1_ESM.zip › 12984_2022_1122_MOESM1_ESM/Table S1.pdf]

Table S1 – Summary of the literature for patient and/or physiotherapist perspectives on exoskeletons.

Citation	User perspective(s)	Device	Setting	Sample conditions or mobility impairment	Design and Methods	Key findings	Theme(s)
Zabel *et al*. (2020)	15 Physiotherapy students	H2 lower limb hard exoskeleton	General clinical setting	Not applicable	Qualitative descriptive study. Focus groups in person in person and online. Participants observed a person being fitted into the H2 suit or it was described to them. They watched videos on a person walking and standing in the device.	Students viewed exoskeletons to be a potentially valuable tool but recognised limitations including the need for informed treatment parameters and appropriate client criteria to guide exoskeleton use for rehabilitation. The study encouraged collaboration between physiotherapist and biomedical engineers.	Safety; One size fits all; Cost of device
Vaughan-Graham *et al.* (2020)	5 persons with stroke and 6 physiotherapists	H2 lower limb hard exoskeleton	Community-based rehab	Persons with stroke	Qualitative interpretive description approach. Semi-structured interviews. Participants used H2 once.	Persons with stroke had concerns that regarding the device: forcing an unnatural walk; its appearance; time to don it on and off; cost. However, they recognised strengths to include being able to walk further. Therapists were most concerned about: device hardware e.g. weight; the device causing compensatory movement; time taken to use; impact on spasticity; safety concerns; and device cost. Similar desire for functions were	Safety; One size fits all; Weight and placement of device; Cost of device; Appearance

						expressed by both therapists and patients, mainly the need for a tailorable gait programme. Physiotherapists highlighted a disconnect between clinical knowledge and the design of devices.	
Read *et al.* (2020)	3 physiotherapists	Ekso GT robotic lower limb exoskeleton	Neurological rehab centre with inpatients and outpatients	Neurological conditions	Exploratory qualitative study using constructivist thematic analysis. Semi-structured interviews. Physiotherapists had undergone level 1 and 2 training with Ekso GT.	All participants expressed exoskeletons increasing their physical work capacity and allowed patients to walk further. The device took time to set up and could cause discomfort if poorly fitted, particularly for those with spasticity; so fitting used valuable therapy time. Cost of device was a concern and it had to be justified. They felt criteria was required for appropriateness of the technology and a guide for use and safety checks.	Safety; One size fits all; Ease of device use
Wolff *et al.* (2014)	354 wheelchair users<br>127 healthcare professionals with experience of working with individuals with mobility impairments	No specific device specified	No exclusion criteria regarding physical abilities. Wheelchair users.	Unspecified conditions, use of wheelchair	Online survey with qualitative and quantitative components. 17 deign features were rated. Content analysis.	The importance of design features were ranked as follows: minimizes risk of falling; purchase cost; comfort; repair and maintenance cost; ease of putting on and taking off the device; range of battery life; ability to walk on uneven surfaces; amount of energy needed for use; ability to carry out daily tasks while standing; portability of the device; ability to toilet; ability to use to get in and out of a car; ability to climb stairs; ability to use without arm crutches; walking speed; length of training to become	Safety; One size fits all; Ease of device use; Weight and placement of device; Cost of device; Appearance

						proficient; overall appearance. Qualitative findings indicated that health and physical benefits, use for activity and access reasons, and psychosocial benefits were important considerations in whether to use or recommend an exoskeleton.	
Bortolle *et al.* (2015)	3 persons with stroke	H2 lower limb exoskeleton	Research setting. Physiotherapist supervising.	Persons with stroke, able to walk without a person's assistance (used walking aids)	Pilot study to evaluate safety and usability of exoskeleton. An assistive gait control algorithm was developed to create a force field along a desired trajectory, only applying torque when patients deviate from the prescribed movement pattern. Participants used the device in 12 sessions over 4 weeks.	Training was well tolerated and no adverse events were reported. The number of steps walked increased across sessions for all participants. No statistical tests are able to be carried out due to small sample size. Donning took <10 minutes, whereas doffing took <2 minutes. Positive feedback received included: “the device is lightweight"; “wearing it is fast and simple"; “I can feel that it helps my knee flexion"; “it is more exciting walking overground with this device than my previous treadmill training with manual assistance" and “I wish I had access to this device when I was in the hospital for inpatient rehabilitation after my stroke". The main negative feedback was: “it felt weird at the first moment and took me some time to get used to it in my first training session, since I have never used a robotic device like this".	Ease of device use
